# Effects of viruses on bacterial functions under contrasting nutritional conditions for four species of bacteria isolated from Hong Kong waters

**DOI:** 10.1038/srep14217

**Published:** 2015-09-25

**Authors:** Hao Liu, Xiangcheng Yuan, Jie Xu, Paul J. Harrison, Lei He, Kedong Yin

**Affiliations:** 1 School of Marine Sciences, Sun Yat-sen University, Guangzhou, China, 510006; 2 Key Laboratory of Marine Resources and Coastal Engineering in Guangdong Province, Guangzhou, China, 510006; 3 Key Laboratory of Tropical Marine Bio-resources and Ecology, South China Sea Institute of Oceanology, Chinese Academy of Sciences, Guangzhou, 510301, China; 4 State Key Laboratory of Tropical Oceanography, South China Sea Institute of Oceanology, Chinese Academy of Sciences, Guangzhou, 510301, China; 5 Department of Earth and Ocean Sciences, University of British Columbia, Vancouver, British Columbia, Canada

## Abstract

Free living viruses are ubiquitous in marine waters and concentrations are usually several times higher than the bacterial abundance. These viruses are capable of lysing host bacteria and therefore, play an important role in the microbial loop in oligotrophic waters. However, few studies have been conducted to compare the role of viruses in regulating bacterial abundance and heterotrophic activities between natural oligotrophic waters and anthropogenic influenced eutrophic waters. In this study, we examined viral effects on bacterial functions of four single bacterial species incubated with natural viral assemblages in seawater samples from eutrophic and oligotrophic waters. The viral-lysis of bacteria was significantly higher in eutrophic than oligotrophic waters. This suggests that viruses were capable of controlling bacterial abundance, respiration and production in the eutrophic waters. Cellular bacterial respiration and production was higher with viruses than without viruses, which was more evident in the oligotrophic waters. These results indicate that viruses can slow down bacterial consumption of oxygen and reduce bacteria-induced eutrophication effects in anthropogenic eutrophic waters, but switch to the role of sustaining the bacterial population when nutrients are limiting. There were bacterial species differences in resisting viral attack, which can influence the dominance and biodiversity of bacterial species in coastal waters.

Viruses play an important ecological role in marine ecosystems^1^,^2^, as viruses are ubiquitous and abundant in the marine environment and are responsible for about 10–50% of the total bacterial mortality^3^,^4^. Bacteria play a major role in global carbon and nutrient cycling. Bacteria can consume 20–60% of the organic carbon produced from primary production in marine ecosystems^5^ by two processes: production of new bacterial biomass (i.e. bacterial production (BP)) and decomposition and remineralization of organic carbon to inorganic carbon (i.e. bacterial respiration (BR)). Bacteria have dual roles in the microbial loop by providing food for micrograzers (microzooplankton, ciliates and heterotrophic dinoflagellates) and also acting as remineralizers of nutrients. In oligotrophic oceans, bacterial biomass may be limited by dissolved organic carbon (DOC) and nutrients and hence, viral lysis of bacteria and the release of cellular lysates can be important in sustaining bacterial production based on the turnover of regenerated DOC and nutrients from cell lysates^6^. Therefore, viral-bacterial interactions play a role in biogeochemical processes and energy fluxes in the ocean^7^,^8^.

Bacterial abundance (BA) is usually controlled by the supply of organic matter and regulates viral abundance. The sequence of peaks in the abundance of phytoplankton - bacteria - viruses is evident during a phytoplankton bloom and subsequent collapse, as reported by Bratbak *et al.* (1990)^9^ and Yager *et al.* (2001)^10^. It is reasonable to hypothesize that in an oligotrophic system, the supply of nutrients limits the production of bacterial organic matter and hence, viruses play an important role in the recycling processes of DOC and nutrients in the microbial loop by lysing bacteria and releasing bacterial lysates. However, in a eutrophic system where organic matter production of phytoplankton or anthropogenic supply of organic matter is high, viruses could play a role similar to grazers and hence, control bacterial abundance, production and respiration.

In natural seawater, viral-bacterial interactions could be affected by multiple factors and processes and it is often difficult to distinguish the viral effect from other effects^11^. To date, there have been few studies that specifically use naturally-occurring single bacterial species to investigate the effects of viruses on bacterial production and respiration. It is important to conduct autecological studies of individual bacterial species, as microbial ecologists have rarely been able to establish links between ecosystem processes and specific bacterial populations^12^. The use of single naturally-occurring bacterial isolates for biogeochemical studies has been limited because only a small percentage of marine bacteria can be cultured. Even fewer experiments have been conducted on how nutrients influence the effects of viruses on bacterial ecological functions using single bacterial species.

In this study, our objective was to examine the effects of viruses on bacterial ecological functions such as bacterial respiration and production of 4 single bacterial species that were isolated from Hong Kong coastal waters. The 4 bacterial species were cultured with the natural viral assemblage in waters from 2 sites with very contrasting nutrient conditions. The water samples were collected from two stations, VM5 in eutrophic Victoria Harbour and PM7 in relatively pristine Port Shelter, having different nutritional conditions. Victoria Harbour receives over 2 × 10^9^ kg of sewage effluent daily from the local sewage system and hence, it represents eutrophic waters^13^ and PM7 is relatively oligotrophic with little influence from sewage^14^. Four single bacterial species belonging to *Proteobacteria* (referred to as S1 to S4, see Methods for details) were used. They were particle-attached and free-living and dominant in Victoria Harbour and were present in oligotrophic waters as well^15^. Our experimental approach is one of the first studies to use single naturally-occurring bacterial species to examine the effects of viruses on bacterial ecological functions under stable laboratory conditions and to explicitly elucidate the processes of viral-bacterial interactions.

## Results

### Conditions of Eutrophic and Oligotrophic Waters

The monthly average of surface temperature, salinity, chlorophyll (Chl *a*), total inorganic nitrogen (NH_4_^+^+NO_3_^−^+NO_2_^−^), PO_4_^3−^ and Si(OH)_4_^−^ over 23 years (1986–2008) (www.epd.gov.hk) showed that VM5 had high Chl *a* (15.3 μg l^−1^) and high nutrients with a 23-year average of 22.6, 0.77 and 21.6 μM for DIN, PO_4_^3−^ and Si(OH)_4_^−^ respectively. In contrast, PM7 had much lower Chl *a* (only 1.7 μg l^−1^) and nutrients. During our sampling time, DIN, PO_4_^3−^ and Si(OH)_4_^−^ was 36, 0.28 and 39 μM, respectively, at VM5, and was 3.4, 0.05 and 16 μM, respectively, at PM7. Hence, we referred to VM5 as eutrophic and PM7 as relatively oligotrophic.

### Viral Effects on Bacterial Abundance

**Figure 1:**
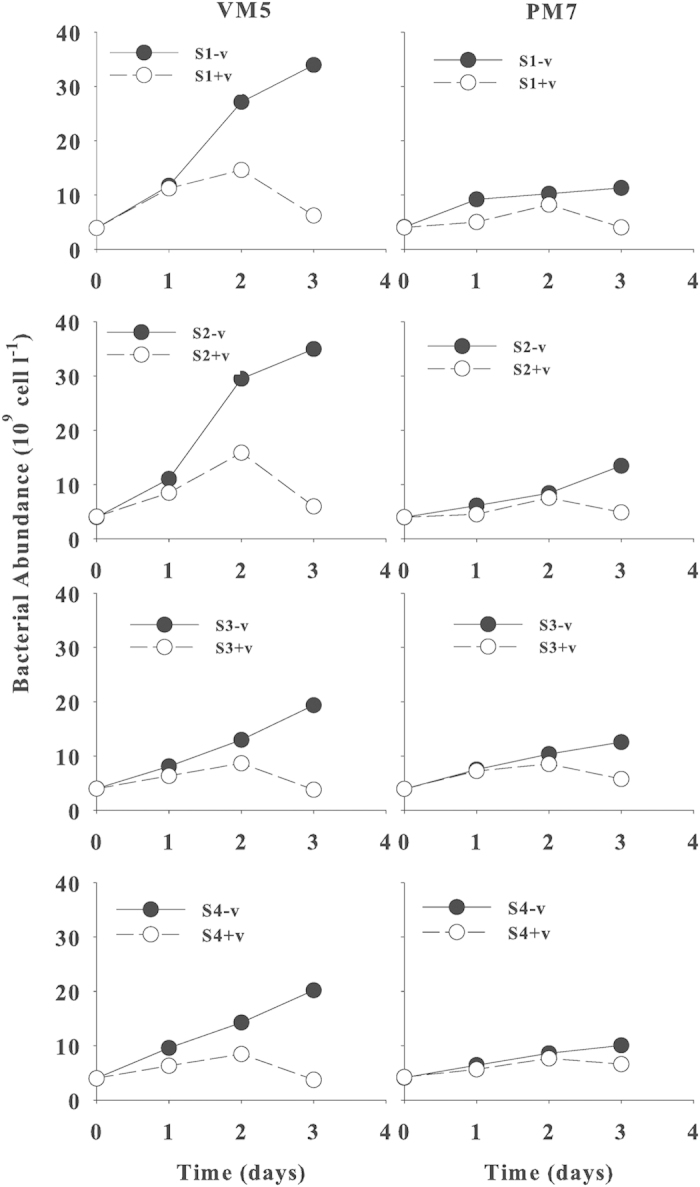
Changes in bacterial abundance (BA) of 4 bacterial species: (S1) *Psychrobacter glacinola* (ICP9), (S2) *Psychrobacter submarinus* (KMM225), (S3) *Marine alpha proteobacterium* (AS-19), (S4) *Alpha proteobacterium* (ISHR1) in the cultures with viruses (+v) (open circles) or without viruses (−v) (filled circles) in waters from Stns VM5 (eutrophic) and PM7 (oligotrophic) during 3 days. Error bars are ± 1 SD and n = 2. The original data are in the supplementary information file.

A change in bacterial abundance with and without viruses (+v and –v) is an important indicator of viral effects on bacteria as viruses use bacterial cells as hosts. Initial bacterial abundance (BA) of the 4 bacterial species was adjusted so that it was similar at 4.0 × 10^9^ cells l^−1^ in all the incubation treatments and therefore, changes in bacterial abundance was mainly due to the presence of viruses. For eutrophic VM5 without viruses, BA increased 5–9 times on day 3 for the 4 species. With viruses, BA of *Gamma-Proteobacteria* had a small increase of only ~1.5 times on day 3, while BA of *Alpha-Proteobacteria* had no significant (*p *< 0.05) increase (Fig. 1). In contrast, for oligotrophic PM7, BA without viruses increased by 2–3 times on day 3 for the 4 species. With viruses, BA increased only 1.2–1.6 times on day 3, except for *Psychrobacter glacinola* (ICP9) which had no significant increase in BA (Fig. 1). Without viruses, the increase in BA was significantly (*p *< 0.01) larger for VM5 than for PM7 on day 3 for all 4 bacterial species. However, the decrease in BA due to the addition of viruses was also larger for VM5 than for PM7, by 80–83% on day 3 for VM5, but by only 34–65% on day 3 for PM7 for all the 4 species. The growth rates of the 4 bacterial species were significantly different between VM5 and PM7.

### Viral Effects on Bacterial Respiration

**Figure 2:**
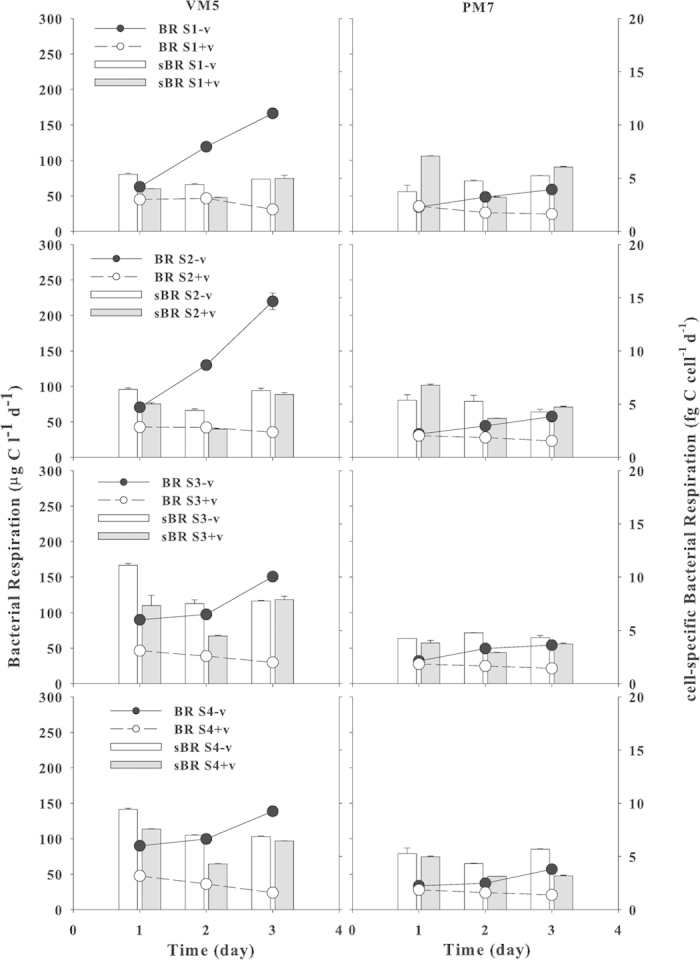
Changes in bacterial respiration (BR) (lines) and cell-specific bacterial respiration (sBR = BR/BA) (vertical bars) for bacterial species S1, S2, S3 and S4 in the cultures with (+v) (open circles) or without viruses (−v) (filled circles) in waters from Stns VM5 and PM7 during 3 days. Gray and white bars are sBR with and without viruses respectively. Error bars are ± 1 SD and *n *= 2.

Bacterial respiration (BR) is an indication of the rate of bacterial heterotrophic activity while utilizing organic matter. Viral lysis of bacterial cells affects BR either by reducing BA or by releasing fresh organic matter (lysates). For eutrophic VM5, BR without viruses was 63–90 μg C l^−1^ d^−1^ on day 1 and increased by 1.5–3.2 times for the 4 species on day 3, while BR with viruses was lower (43–48 μg C l^−1^ d^−1^) on day 1 and decreased further by 17–50% on day 3 (Fig. 2). In contrast, for oligotrophic PM7, BR without viruses was 32–34 μg C l^−1^ d^−1^ on day 1 and increased by 1.5–2.0 times on day 3, while BR with viruses was 26–35 μg C l^−1^ d^−1^ on day 1 and decreased by 22–31% on day 3. BR without viruses was significantly (*p *< 0.01) higher for VM5 than for PM7. However, cell-specific BR (sBR) did not show the same trend as BR. Without viruses, sBR for VM5 remained similar (5 fg C cell^−1^ d^−1^) on days 1–3 for S1 and S2, but almost doubled (9–11 fg C cell^−1^ d^−1^) on day 1 for S3 and S4. The addition of viruses caused a decrease in sBR on day 1, but not on day 3 for S3 and S4 (Fig. 2). In contrast, for PM7, sBR without viruses was lower (3–5 fg C cell^−1^ d^−1^) than with viruses for all 4 species on day 1 with little change on day 3. The addition of viruses resulted in an increase in sBR for S1 and S2 on day 1, but not for S3 and S4. On day 3, comparing the two treatments with (+v) and without (–v) viruses, sBR without viruses was significantly (*p *< 0.01) higher than with viruses for VM5, whereas there was no significant (*p *> 0.05) difference in sBR for PM7. All cell-specific BR rates with viruses showed a decrease on day 2.

### Viral Effects on Bacterial Production

**Figure 3:**
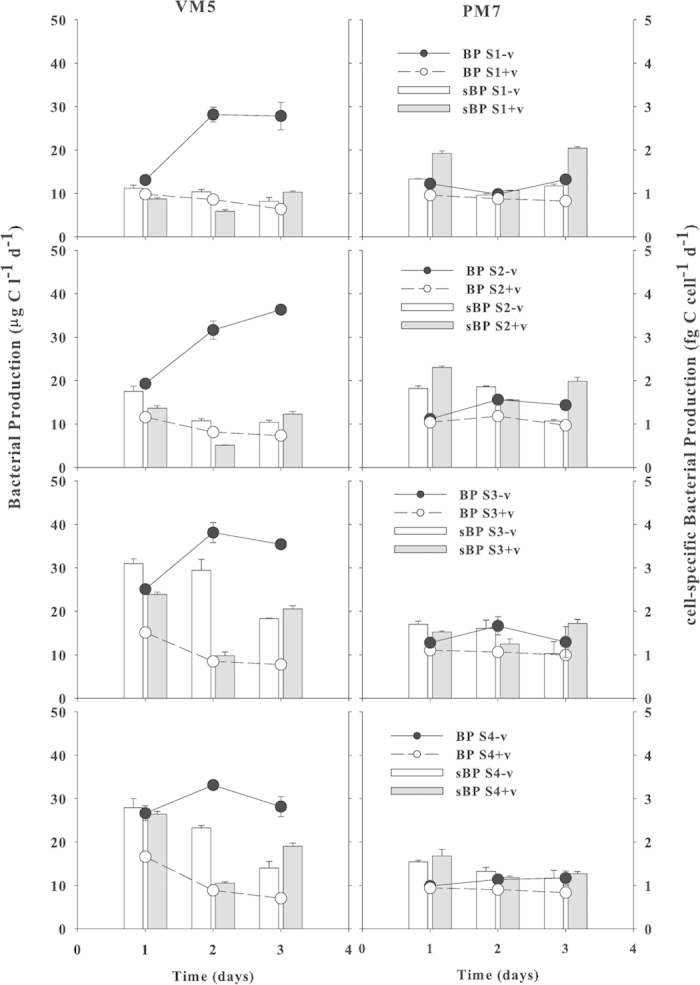
Changes in bacterial production (BP) (lines) and cell-specific bacterial production (sBP = BP/BA) (vertical bars) for 4 bacterial species S1, S2, S3 and S4 in the cultures with (+v) (open circles) or without viruses (−v) (filled circles) in waters from Stns VM5 and PM7 during 3 days. Gray and white bars are sBP with and without viruses respectively. Error bars are ± 1 SD and n = 2.

Bacterial production (BP) reflects bacterial growth, which can be a function of BA, and hence, viral lysis can affect BP. For eutrophic VM5, BP without viruses was 13–27 μg C l^−1^ d^−1^ on day 1 and increased (1.2–2.1 times) for the 4 bacterial species on day 3, while BP with viruses was lower (10–17 μg C l^−1^ d^−1^) on day 1 and decreased further by 35–58% on day 3 (Fig. 3). For oligotrophic PM7, BP without viruses was lower (10–13 μg C l^−1^ d^−1^) than that for VM5 on days 1–3 for all 4 bacterial species. In contrast, BP with viruses (9–11 μg C l^−1^ d^−1^ on day 1) only decreased by 7–14% on day 3. For eutrophic VM5, sBP without viruses was higher than sBP with viruses. For VM5, the differences in sBR between the treatments with and without viruses was the largest on day 2 (significant at *p *< 0.05) , but not significantly (*p *> 0.05) different on day 1 and 3, except for S3 on day 1 and S4 on day 3. For PM7, sBP with and without viruses had a similar temporal fluctuation with a significant drop on day 2. In contrast to VM5, the addition of viruses to PM7 caused a significantly large increase in sBP on day 1 and 3 among all the 4 bacterial species (except for S3 on day 1, and for S1 on day 2) (Fig. 3).

There were bacterial species differences in sBP. Without viruses, sBP was higher for S3 and S4 (*Alpha-Proteobacteria*) than for S1 and S2 (*Gamma-Proteobacteria*) for VM5, whereas for PM7, sBP without viruses was not significantly (*p *> 0.05) different among the 4 species. However, the addition of viruses resulted in higher sBP for S1 and S2 (*Gamma-Proteobacteria*) than for S3 and S4 (*Alpha-Proteobacteria*) on days 1 and 3, and also higher than the same two species for VM5. For PM7, there was a contrasting difference in sBP between the 2 treatments with viruses (1.3–2.0 fg C cell^−1^ d^−1^) and without viruses (~1.1 fg C cell^−1^ d^−1^) on day 3. The sBP with viruses on day 2 was the lowest for both VM5 and PM7. For VM5, sBP of *Alpha-Proteobacteria* (S3 and S4) was significantly (*p *< 0.01) higher than for *Gamma-Proteobacteria* (S1 and S2) with or without viruses (Fig. 3).

### Viral Effects on Bacterial Growth Efficiency

**Figure 4:**
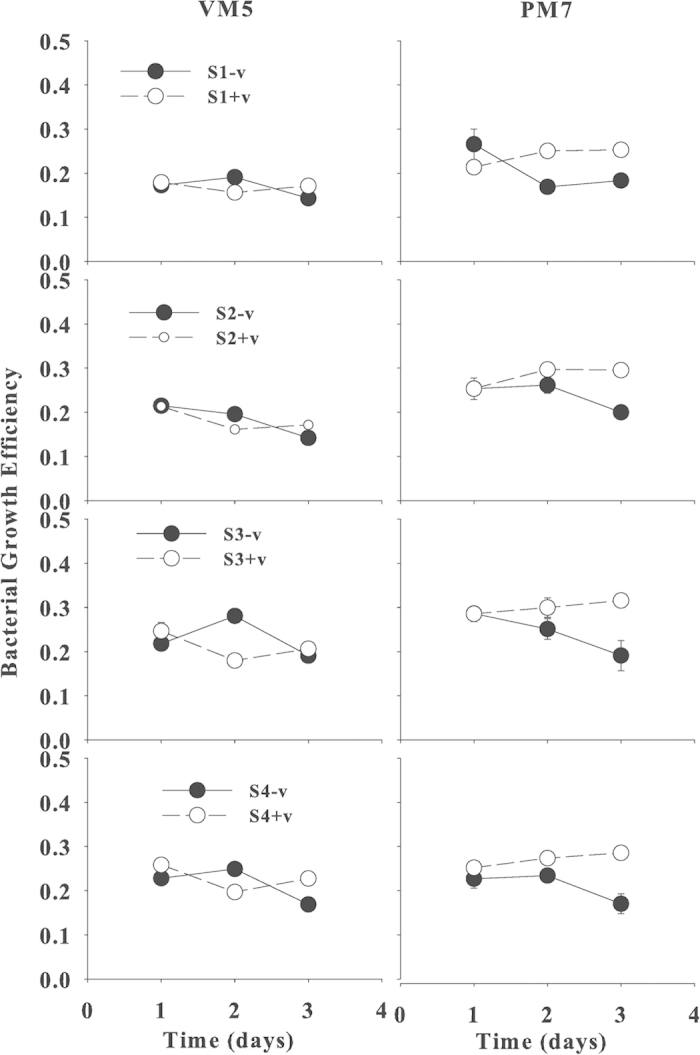
Temporal changes in bacterial growth efficiency (BGE) of 4 bacterial species S1, S2, S3 and S4 in the cultures with (+v) (open circles) or without viruses (−v) (filled circles) in waters from Stns VM5 and PM7 during 3 days. Error bars are ± 1 SD and often smaller than the symbol size and n = 2. The original data are in the supplementary information file.

Bacterial growth efficiency (BGE) without viruses eventually decreased for both VM5 and PM7 on day 3. However, BGE responded differently to the addition of viruses. For VM5, there was no significant (*p *> 0.05) difference in BGE with or without viruses, however, for PM7, BGE with viruses increased significantly (*p *< 0.01) relative to without viruses (Fig. 4).

## Discussion

This study showed that viruses affected bacterial abundance, respiration and production as well as bacterial growth efficiency, and demonstrated the different ecological roles of viruses in eutrophic and oligotrophic ecosystems. There were also bacterial species differences in the bacterial responses to the presence or absence of viruses.

Studies of viral effects on a single bacterial strain are rare, but such studies are necessary to elucidate specific processes of viral effects on bacteria. In addition, natural seawater contains a viral community, which is an important mediator of genetic exchange^16^. Using a natural viral assemblage has the advantage of containing viruses capable of infecting a single bacterial species. Our study clearly showed that bacterial cells were infected within a day, which caused a decrease in abundance by up to 34–65% in a single bacterial species due to viral lysis. These results were consistent with other studies using natural seawater which showed that 20–50% of the bacterial biomass was lost daily due to viral lytic infection^8^. In planktonic systems, 5–30% of the heterotrophic bacteria and cyanobacteria are infected by viruses at any time, and viruses can lyse a substantial fraction (4–50%) of the daily bacterial and cyanobacterial production^1^,^17^. The viral-induced mortality is on average as significant as grazing of bacteria by protists^18^, although the relative importance might vary spatially and temporally with different environmental conditions^19^,^20^.

Virus-caused bacterial mortality is a function of bacterial abundance^18^. Steward *et al.* (1996)^21^ and Weinbauer (2004)^4^ suggested that the viral effect on bacterial abundance was probably larger in eutrophic than oligotrophic waters. In our study, the viral lysis of bacteria was magnified in the eutrophic waters at VM5, as shown by a decrease in BA, BR and BP by 80–83%, 17–50% and 35–58%, respectively, for the 4 bacterial species on day 3. This finding indicates that viruses can exert an analogous ‘top-down’ control of BA, BP and BR for bacteria in eutrophic environments. Viral lysis reduced BR and BP, which could slow down the consumption of dissolved oxygen and reduce bacterial decomposition of organic matter. This has important implications for how viruses could influence the formation of hypoxia and for increasing the export of organic matter from eutrophic bays to open waters.

Viruses can influence bacterial respiration and production^22^,^23^. In our study, the addition of viruses resulted in a decrease in BR by 17–50% in all 4 bacterial species for VM5 and by 22–31% for PM7 on day 3 compared with day 1. Cellular BR (sBR) responded differently between eutrophic VM5 and oligotrophic PM7: no significant difference (*p *< 0.01) for the former on day 3, but a significant increase for *Gamma-Proteobacteria* by 11–16% and a decrease by 14–44% for *Alpha-Proteobacteria* for the latter. Our findings agree with a previous study by Xu *et al.* (2013)^24^ who used a natural assemblage of bacteria for the same stations and they reported that sBR was higher at PM7 than VM5, in agreement with a similar previous study by Bonilla-Findji *et al.* (2008)^25^.

The addition of viruses caused BP to decrease by 35–58% in the 4 bacterial species in the eutrophic waters of VM5, while the decrease was much less in oligotrophic PM7, by only 7–14% (on day 3). This was in agreement with other studies^6^,^25^,^26^. The difference in nutrient concentrations between VM5 and PM7 might be responsible for the difference in the viral effect on sBP: a larger increase for the oligotrophic waters at PM7 than eutrophic water at VM5 in which sBP on day 3 was not significantly (*p *< 0.01) different from day 1.

BGE is considered to be a good indicator of carbon flow through bacteria^27^. Our results appeared to indicate that more carbon flowed through bacterial production with the addition of viruses in the oligotrophic water, as BGE in virus-added treatments was higher (0.25–0.32) for PM7 than (0.17–0.23) for VM5 on day 3. According to Fuhrman (1999)^7^, when viruses lyse bacterial cells, the lysed products are available to other bacterial cells. This represents a semi-closed trophic loop, namely the lysed bacterial biomass being consumed by other bacteria via regeneration of DOC and inorganic nutrients. Such a viral effect was magnified in the oligotrophic water of PM7. This was consistent with other observations of natural assemblages of bacteria in other oligotrophic waters^28^,^29^.

**Figure 5:**
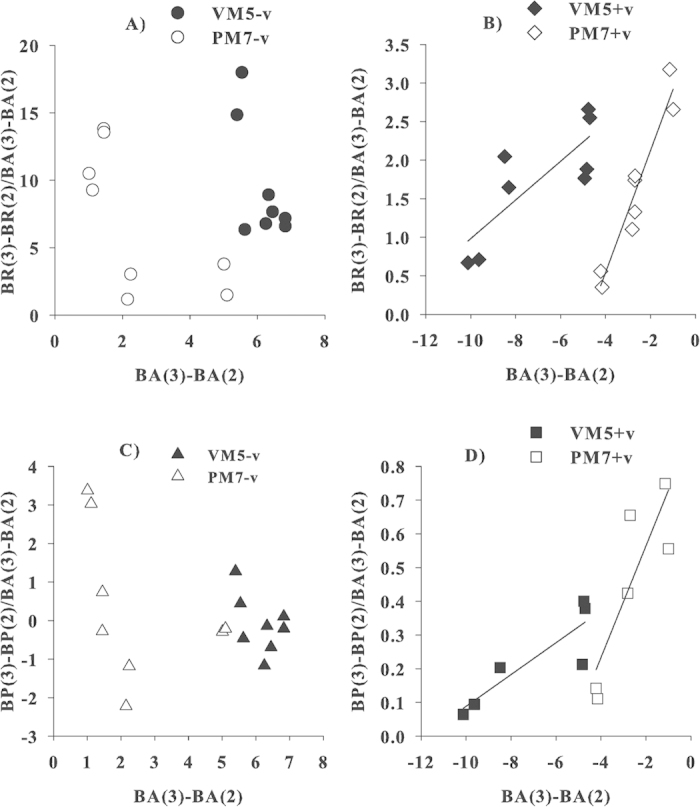
Correlations between changes in bacterial abundance from day 2 to 3 and the corresponding, cell specific bacterial respiration and production for VM5 and PM7. Assuming the concentration of the dead bacterial cells was equal to ΔBA_(3)–(2)_ using BA on day 3 minus BA on day 2, the corresponding change in BR and BP was ΔBR_(3)–(2)_ and ΔBP_(3)–(2)_ using BP and BR on day 3 minus day 2. (**A**) ΔBR without viruses (VM5, black circles; PM7, white circles), no significant linear relationships, (**B**) ΔBR with viruses (VM5, black diamonds; PM7, white diamonds), significant linear relationships are ΔBR = 0.25 × ΔBA + 3.49, *r*^*2 *^= 0.66, *p *< 0.05, n = 8 for VM5; ΔBR = 0.79 × ΔBA + 3.71, *r*^*2 *^= 0.92, *p *< 0.05, n = 8 for PM7, (**C**) ΔBP without viruses (VM5, black triangles; PM7, white triangles), no significant linear relationships, and D) ΔBP with viruses (VM5, black squares; PM7, white squares), significant linear relationships are ΔBP = 0.05 × ΔBA + 0.56, *r*^*2 *^= 0.77, *p *< 0.05, n = 6 for VM5 and ΔBR = 0.17 × ΔBA + 0.88, *r*^*2 *^= 0.76, *p *< 0.05, n = 6 for PM7.

There was a possibility that viral lysis of bacterial cells resulted in higher supply to bacterial carbon demand (BCD) in the oligotrophic water than in eutrophic waters. Assuming that the lysed cells were used to support bacterial growth, the concentration of dead cells should result in the corresponding increase in cell specific BR and BP. Indeed, without viruses, no significant relationship was found between the amount of dead cells [ΔBA_(3)–(2)_] versus the amount of DO respired per cell [ΔBR_(3)–(2)_/ΔBA_(3)–(2)_] (Fig. 5A), and versus the amount of bacterial carbon produced per cell [ΔBP_(3)–(2)_/ΔBA_(3)–(2)_] (Fig. 5C) in either the eutrophic or oligotrophic waters. However, the presence of viruses resulted in a significant relationship between ΔBA_(3)-(2)_ and ΔBR_(3)–(2)_/ ΔBA_(3)–(2)_ (Fig. 5B) and between ΔBA_(3)–(2)_ and ΔBP_(3)–(2)_/ΔBA_(3)–(2)_ (Fig. 5D) in both the eutrophic and oligotrophic waters. This strongly indicates that the lysed bacterial cells were used to support the growth and respiration of the remaining bacterial cells. Furthermore, the slope of the relationship was steeper for the oligotrophic than eutrophic waters. This indicated that viral lysis of bacterial cells in the nutrient-limited waters was more efficient in supporting bacterial activities such as BR and BP than those in the nutrient-rich water. Other studies using natural assemblages also found that lysed products could be a dominant organic source for meeting bacterial carbon demand^30^. Noble and Fuhrman (1999)^26^ reported that products from viral lysis turned over rapidly, especially in P-limited oligotrophic waters and the DOM released during lysis could stimulate the non-infected bacterial populations^31^.

**Figure 6:**
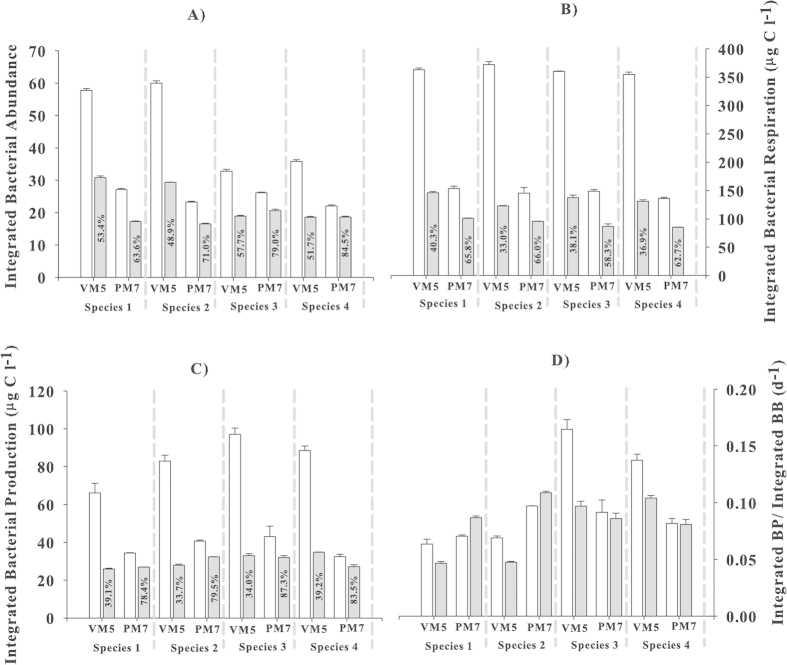
Comparison between the 4 bacterial species (see the legend in Fig. 1 for the names) in: (A) 3 day-integrated bacterial abundance (IBA), (B) integrated bacterial respiration (IBR), (C) integrated bacterial production (IBP) and (D) the ratios of IBP to integrated bacterial biomass (IBB). Open bars represent the virus-free treatment and gray bars represent the virus-added treatment. The % value within the gray bar is the percent reduction of those parameters compared to the virus-free treatment. Error bars are ± 1 SD and n = 2.

Turnover rate (d^−1^) of bacterial cells and viruses can be an indicator of the regeneration of nutrients. The ratio of time integrated (3 days) bacterial production (IBP) to time integrated bacterial biomass (IBB) in our study, which is basically the carbon turnover rate, showed that the presence of viruses increased the turnover rate for the oligotrophic PM7 waters more than for eutrophic VM5, except for species 3 (Fig. 6D). This suggested that viral lysis in oligotrophic waters plays a more important role in regenerating nutrients and bacterial production.

Bacterial diversity plays an important role in ecosystem functions^32^. In general, there are two principal mechanisms: the ‘complementarity mechanism’ and the ‘selection mechanism’, which explain how species diversity is maintained^33^. A more recent study was conducted on the contribution of species richness and composition to bacterial ecological services and found effects for both mechanisms^34^. Our results revealed the effects of a natural viral assemblage on individual bacterial species and clearly demonstrated differences among bacterial species in their ecological functions without viruses and their responses to viral attacks. Since the initial values were similar among all the treatments, integrating BA, BR and BP (IBA, IBR and IBP) over 3 days represents the total species differences in BA, BR and BP. In eutrophic waters (VM5), IBA without viruses was much higher for bacterial S1 and S2 than for S3 and S4, and the reduction in IBA by the addition of viruses was significantly different among all 4 species (Fig. 6A). IBP without viruses was also different among the 4 bacterial species for VM5 (Fig. 6C), although IBR without viruses was not significantly different (Fig. 6B). In oligotrophic waters (PM7), species differences in IBA, IBR and IBP without viruses were not as large as in the eutrophic water (VM5). However, the viral-induced reduction of IBA, IBR and IBP was much smaller for each species in oligotrophic (PM7) than eutrophic waters (VM5). IBA, IBR and IBP decreased to 47, 63 and 64% respectively, in eutrophic waters after viruses were added, while in oligotrophic waters, the decrease in IBA, IBR and IBP was much smaller (by 35, 37 and 18% respectively). This indicates that external factors such as substrate supply likely drive all species, while species differences exert their dominance based on their responses to a particular external force. In our study, the bacterial response to nutrient supply determined their dominance between eutrophic and oligotrophic waters and the bacterial resistance to viral attack could play a role in their dominance within a water body. As Zhang *et al.* (2007)^35^ observed, the addition of viruses resulted in a decrease in bacterial abundance, but a significant increase in the bacterial species diversity. This suggests that viruses could serve a role similar to the grazing role of protists in maintaining bacterial diversity.

## Methods

### Experimental Waters

Water samples were collected on July 5 2008 at 1 m from two stations: eutrophic VM5 in Victoria Harbour and oligotrophic PM7 in the Port Shelter. Victoria Harbour receives over 2 × 10^9^ kg of sewage effluent daily from the local sewage outfall and hence, it represents a eutrophic water body. In contrast, PM7 is relatively oligotrophic with little influence from sewage, and it has the lowest dissolved inorganic N concentrations during July in Hong Kong waters^14^. Initial concentrations of bacteria and viruses as well as nutrients were measured.

### Virus-Free Water

Water samples were filtered through 1-μm pore-sized filter (Isopore ATTP) and the filtrate was filtered twice through a 0.2-μm cartridge to obtain bacteria-free seawater. The bacteria-free seawater was filtered through a 100-kDa cut–off polyethersulfone membrane cartridge (Prep/scale-THH, CDUF002TH; Millipore) to obtain virus-free seawater (the filtrate) and then the viruses retained on the cartridge were gently washed off to obtain the virus-concentrated seawater. The initial viral abundance was adjusted to achieve a similar abundance of 5.7 ± 0.77 × 10^10^ and 5.8 ± 0.73 × 10^10^ cells l^-1^, respectively, for VM5 and PM7 so that the observed differences would not be due to different initial viral concentrations the results would be independent of the initial viral concentration.

### Single Bacterial Species

Four bacterial species which were isolated from Hong Kong coastal waters included *Psychrobacter glacinola* (ICP9), *Psychrobacter submarinus* (KMM225), *Marine alpha proteobacterium* (AS-19), and *Alpha proteobacterium* (ISHR1) and were abbreviated as S1, S2, S3 and S4, respectively. They were isolated and collected by the Coastal Marine Laboratory of Hong Kong University of Science and Technology (http://www.cml.ust.hk/research_center.html). The former two species belong to *Gamma-Proteobacteria* and the latter two belong to *Alpha-Proteobacteria*^35^. These bacterial species were grown at 20 °C in the dark with marine broth 2216 (Difco) and mid-exponential phase cultures were used as the inocula for the incubation experiments.

### Experimental Design

An inoculum of the bacterial culture was transferred to the filtered seawater samples with and without viruses in pre-sterilized 1-liter glass bottles, with the initial concentrations of bacteria and viruses being adjusted so that they were equal to the *in situ* concentrations of the samples at the time of the sampling. All bottles were incubated in the dark for 3 days and an environmental chamber with constant temperature control was used to maintain the *in situ* temperature (28 ± 2 °C). There were 2 treatments: a bacterial culture with viruses (+v) and without viruses (−v). Replicate errors were found to be small as reported in Xu *et al.* (2013)^24^ and hence, we used duplicates for each treatment. During the 3 day incubation, samples for analysis of bacterial abundance (BA), bacterial production (BP) and bacterial respiration (BR) were taken daily.

*Determination of BA, VA and BP—*Bacterial abundance (BA) was determined by the direct count method^36^. Integrated bacterial biomass (IBB) was estimated using integrated bacterial abundance (IBA) and a conversion factor of 20 fg carbon per cell^37^.

Viral abundance was determined based on the method by Noble and Fuhrman (1998)^38^.

Bacterial production (BP) was measured using ^3^H-leucine following the JGOFS protocols^39^. The incorporated ^3^H was determined using a Perkin-Elmer Wallac (Beckman® 1414 CA/LL) liquid scintillation counter. BP was calculated using the empirical conversion factor of 3 kg C mol leucine^−1^ 40. Cell-specific bacterial respiration and production (sBR and sBP) were calculated by normalizing BR and BP to BA, respectively.

Dissolved oxygen (DO) was determined in duplicate by the Winkler titration method following the JGOFS protocols^39^.

For BR measurements, six 60 ml BOD bottles were used and BR was the difference in DO between the initial and final concentration over the 24 h incubation and was expressed in carbon units assuming a respiratory quotient of 1^41^. Bacterial growth efficiency (BGE) was calculated using the following equation:


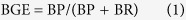


Statistical analyses were performed using SPSS software. The significance of the effects of the virus additions in the treatments was assessed by using an analysis of variance followed by a means comparison (t-test) between the treatments at a significance level of *p *< 0.05. The error bars represent a pooled sample standard deviation of the means.

## Additional Information

**How to cite this article**: Liu, H. *et al.* Effects of viruses on bacterial functions under contrasting nutritional conditions for four species of bacteria isolated from Hong Kong waters. *Sci. Rep.* **5**, 14217; doi: 10.1038/srep14217 (2015).

## Supplementary Material

Supplementary Information
