# Supplementary material for: Effects of viruses on bacterial functions under contrasting nutritional conditions for four species of bacteria isolated from Hong Kong waters
Source: Sci Rep. 2015 Sep 25;5:14217. doi: 10.1038/srep14217 (PMC4585901; doi:10.1038/srep14217)
Supplement: Supplementary Information [file srep14217-s1.doc]

Effects of viruses on bacterial functions under contrasting nutritional conditions for four isolated species of bacteria from Hong Kong waters

[Hao Liu](#OLE_LINK74), ^1, 2^ [Xiangcheng Yuan](#OLE_LINK77),^3^ [Jie Xu](#OLE_LINK78),^4^ [Paul J. Harrison](#OLE_LINK76),^5^ [Lei He](#OLE_LINK79), ^1, 2^and [Kedong Yin](#OLE_LINK75), ^1, 2,*^

**Supplementary data**

S1, S2, S3 and S4 represent *Psychrobacter glacinola* (ICP9)，*Psychrobacter submarinus* (KMM225), *Marine alpha proteobacterium* (AS-19), and *Alpha proteobacterium* (ISHR1), respectively. The symbols +v and –v represent the treatments with viruses and without viruses. D0, D1, D2 and D3 represent day 0, 1, 2 and 3, respectively. BA, BR and BP represent bacterial abundance, respiration and production, respectively. sBR (sBR = BR/BA) and sBP (sBP = BP/BA) represent cell-specific bacterial respiration and production. BGE [BGE = BP/(BP+BR)] represent bacterial growth efficiency. IBA, IBR, IBR and IBB represent 3 day-integrated bacterial abundance, respiration, production and biomass, respectively. SD represents standard deviations. The unit of BA is 10^9^ cell l^-1^ and the unit of BR and BP is all μg C l^-1^ d^-1^. The unit of sBR and sBP is all fg C l^-1^ d^-1^. The value of SD is ±. The unit of IBR, IBP is all μg C l^-1^.

| BA | VM5 | | | | | | | | | | | | PM7 | | | | | | | | | | | |
| --- | --- | --- | --- | --- | --- | --- | --- | --- | --- | --- | --- | --- | --- | --- | --- | --- | --- | --- | --- | --- | --- | --- | --- | --- |
|  | S1  -v | S1  -v | SD | S2  -v | S2  -v | SD | S3  -v | S3  -v | SD | S4  -v | S4  -v | SD | S1  -v | S1  -v | SD | S2  -v | S2  -v | SD | S3  -v | S3  -v | SD | S4  -v | S4  -v | SD |
| D0 | 3.9 | 3.9 | 0 | 4.0 | 3.9 | 0.05 | 4.0 | 4.0 | 0 | 4.0 | 4.0 | 0 | 4.0 | 4.1 | 0.05 | 3.9 | 4.0 | 0.05 | 4.0 | 3.9 | 0.05 | 4.2 | 4.1 | 0.05 |
| D1 | 11.8 | 11.6 | 0.1 | 10.9 | 11.1 | 0.1 | 8.2 | 8.0 | 0.1 | 9.6 | 9.5 | 0.05 | 9.1 | 9.3 | 0.1 | 6.1 | 6.2 | 0.05 | 7.6 | 7.4 | 0.1 | 6.5 | 6.3 | 0.1 |
| D2 | 27.3 | 26.9 | 0.2 | 29.9 | 29.1 | 0.4 | 13.2 | 12.7 | 0.25 | 14.3 | 14.2 | 0.05 | 10.3 | 10.1 | 0.1 | 8.3 | 8.5 | 0.1 | 10.4 | 10.3 | 0.05 | 8.7 | 8.5 | 0.1 |
| D3 | 34.2 | 33.7 | 0.25 | 35.4 | 34.5 | 0.45 | 19.6 | 19.2 | 0.2 | 20.6 | 19.8 | 0.4 | 11.2 | 11.4 |  | 13.4 | 13.6 | 0.1 | 12.6 | 12.5 | 0.05 | 10.1 | 9.9 | 0.1 |
| BA | S1  +v | S1  +v | SD | S2  +v | S2  +v | SD | S3  +v | S3  +v | SD | S4  +v | S4  +v | SD | S1  +v | S1  +v | SD | S2  +v | S2  +v | SD | S3  +v | S3  +v | SD | S4  +v | S4  +v | SD |
| D0 | 3.9 | 3.9 | 0 | 4.0 | 4.2 | 0.1 | 4.0 | 3.9 | 0.05 | 4.0 | 3.9 | 0.05 | 4.0 | 40. | 0 | 3.9 | 4.0 | 0.05 | 4.0 | 3.9 | 0.05 | 4.2 | 4.2 | 0 |
| D1 | 11.3 | 11.1 | 0.1 | 8.6 | 8.4 | 0.1 | 6.4 | 6.3 | 0.05 | 6.4 | 6.1 | 0.15 | 5.1 | 4.9 | 0.1 | 4.5 | 4.6 | 0.05 | 7.3 | 7.1 | 0.1 | 5.6 | 5.7 | 0.05 |
| D2 | 14.8 | 14.4 | 0.2 | 15.7 | 16.0 | 0.15 | 8.7 | 8.6 | 0.05 | 8.5 | 8.4 | 0.05 | 8.3 | 8.2 | 0.05 | 7.5 | 7.6 | 0.05 | 8.6 | 8.4 | 0.1 | 7.5 | 7.8 | 0.15 |
| D3 | 6.3 | 6.1 | 0.1 | 6.1 | 5.9 | 0.1 | 3.8 | 3.8 | 0 | 3.7 | 3.7 | 0 | 4.1 | 3.9 | 0.1 | 4.8 | 4.9 | 0.05 | 5.9 | 5.6 | 0.15 | 6.5 | 6.6 | 0.05 |

| BR | VM5 | | | | | | | | | | | | PM7 | | | | | | | | | | | |
| --- | --- | --- | --- | --- | --- | --- | --- | --- | --- | --- | --- | --- | --- | --- | --- | --- | --- | --- | --- | --- | --- | --- | --- | --- |
|  | S1  -v | S1  -v | SD | S2  -v | S2  -v | SD | S3  -v | S3  -v | SD | S4  -v | S4  -v | SD | S1  -v | S1  -v | SD | S2  -v | S2  -v | SD | S3  -v | S3  -v | SD | S4  -v | S4  -v | SD |
| D1 | 64 | 61 | 1.5 | 71 | 70 | 0.5 | 90 | 90 | 0 | 90 | 90 | 0 | 38 | 30 | 4.0 | 30 | 35 | 2.5 | 32 | 32 | 0 | 32 | 36 | 2.0 |
| D2 | 119 | 120 | 0.5 | 129 | 131 | 1.0 | 96 | 99 | 1.5 | 100 | 99 | 0.5 | 47 | 49 | 2.0 | 41 | 48 | 3.5 | 50 | 50 | 0 | 38 | 37 | 0.5 |
| D3 | 168 | 165 | 1.5 | 228 | 212 | 8.0 | 153 | 148 | 2.5 | 142 | 135 | 3.5 | 58 | 60 | 1.0 | 60 | 56 | 2.0 | 56 | 52 | 2.0 | 58 | 56 | 1.0 |
| BR | S1  +v | S1  +v | SD | S2  +v | S2  +v | SD | S3  +v | S3  +v | SD | S4  +v | S4  +v | SD | S1  +v | S1  +v | SD | S2  +v | S2  +v | SD | S3  +v | S3  +v | SD | S4  +v | S4  +v | SD |
| D1 | 45 | 44 | 0.5 | 42 | 43 | 0.5 | 43 | 50 | 3.5 | 49 | 47 | 1.0 | 36 | 35. | 0.5 | 31 | 31 | 0 | 29 | 26 | 1.5 | 28 | 28 | 0 |
| D2 | 47 | 45 | 1.0 | 42 | 42 | 0 | 40 | 38 | 1.0 | 37 | 36 | 0.5 | 26 | 26 | 0 | 28 | 28 | 0 | 25 | 24 | 0.5 | 24 | 24 | 0 |
| D3 | 30 | 32 | 1.0 | 35 | 35 | 0 | 31 | 29 | 1.0 | 24 | 24 | 0 | 25 | 24 | 0.5 | 23 | 23 | 0 | 22 | 21 | 0.5 | 21 | 21 | 0 |

| sBR | VM5 | | | | | | | | | | | | PM7 | | | | | | | | | | | |
| --- | --- | --- | --- | --- | --- | --- | --- | --- | --- | --- | --- | --- | --- | --- | --- | --- | --- | --- | --- | --- | --- | --- | --- | --- |
|  | S1  -v | S1  -v | SD | S2  -v | S2  -v | SD | S3  -v | S3  -v | SD | S4  -v | S4  -v | SD | S1  -v | S1  -v | SD | S2  -v | S2  -v | SD | S3  -v | S3  -v | SD | S4  -v | S4  -v | SD |
| D1 | 5.4 | 5.3 | 0.05 | 6.5 | 6.3 | 0.1 | 11.0 | 11.2 | 0.1 | 9.3 | 9.5 | 0.1 | 4.2 | 3.3 | 0.45 | 5.0 | 5.7 | 0.35 | 4.3 | 4.3 | 0 | 4.9 | 5.6 | 0.35 |
| D2 | 4.3 | 4.5 | 0.1 | 4.3 | 4.5 | 0.1 | 7.8 | 7.3 | 0.25 | 7.0 | 7.0 | 0 | 4.7 | 4.8 | 0.05 | 4.9 | 5.7 | 0.4 | 4.8 | 4.8 | 0 | 4.4 | 4.3 | 0.05 |
| D3 | 4.9 | 4.9 | 0 | 6.4 | 6.1 | 0.15 | 7.8 | 7.7 | 0.05 | 6.9 | 6.8 | 0.05 | 5.2 | 5.2 | 0 | 4.5 | 4.1 | 0.2 | 4.5 | 4.2 | 0.15 | 5.7 | 5.7 | 0 |
| sBR | S1  +v | S1  +v | SD | S2  +v | S2  +v | SD | S3  +v | S3  +v | SD | S4  +v | S4  +v | SD | S1  +v | S1  +v | SD | S2  +v | S2  +v | SD | S3  +v | S3  +v | SD | S4  +v | S4  +v | SD |
| D1 | 4.0 | 4.0 | 0 | 5.0 | 5.1 | 0.05 | 6.7 | 8.0 | 0.65 | 7.5 | 7.6 | 0.05 | 7.0 | 7.1 | 0.05 | 6.9 | 6.7 | 0.1 | 4.0 | 3.6 | 0.2 | 5.0 | 4.9 | 0.05 |
| D2 | 3.2 | 3.2 | 0 | 2.7 | 2.6 | 0.05 | 4.5 | 4.4 | 0.05 | 4.3 | 4.3 | 0 | 3.2 | 3.2 | 0 | 3.7 | 3.7 | 0 | 2.9 | 2.9 | 0 | 3.1 | 3.1 | 0 |
| D3 | 4.8 | 5.2 | 0.2 | 5.8 | 6.0 | 0.1 | 8.1 | 7.7 | 0.2 | 6.5 | 6.4 | 0.05 | 6.0 | 6.1 | 0.05 | 4.8 | 4.7 | 0.05 | 3.7 | 3.8 | 0.05 | 3.2 | 3.1 | 0.05 |

| BP | VM5 | | | | | | | | | | | | PM7 | | | | | | | | | | | |
| --- | --- | --- | --- | --- | --- | --- | --- | --- | --- | --- | --- | --- | --- | --- | --- | --- | --- | --- | --- | --- | --- | --- | --- | --- |
|  | S1  -v | S1  -v | SD | S2  -v | S2  -v | SD | S3  -v | S3  -v | SD | S4  -v | S4  -v | SD | S1  -v | S1  -v | SD | S2  -v | S2  -v | SD | S3  -v | S3  -v | SD | S4  -v | S4  -v | SD |
| D1 | 17 | 15 | 1.0 | 24 | 22 | 1.0 | 30 | 31 | 0.5 | 31 | 33 | 1.0 | 14 | 15 | 0.5 | 14 | 13 | 0.5 | 16 | 15 | 0.5 | 12 | 11 | 0.5 |
| D2 | 35 | 32 | 1.5 | 40 | 36 | 2.0 | 44 | 48 | 2.0 | 41 | 39 | 1.0 | 12 | 12 | 0 | 18 | 19 | 0.5 | 22 | 18 | 2.0 | 13 | 14 | 0.5 |
| D3 | 36 | 30 | 3.0 | 44 | 43 | 0.5 | 43 | 42 | 0.5 | 32 | 36 | 2.0 | 16 | 16 | 0 | 17 | 18 | 0.5 | 19 | 13 | 3.0 | 14 | 15 | 0.5 |
| BP | S1  +v | S1  +v | SD | S2  +v | S2  +v | SD | S3  +v | S3  +v | SD | S4  +v | S4  +v | SD | S1  +v | S1  +v | SD | S2  +v | S2  +v | SD | S3  +v | S3  +v | SD | S4  +v | S4  +v | SD |
| D1 | 12 | 12 | 0 | 14 | 14 | 0 | 18 | 18 | 0 | 19 | 20 | 0.5 | 11 | 12 | 0.5 | 12 | 12 | 0 | 13 | 13 | 0 | 12 | 11 | 0.5 |
| D2 | 10 | 11 | 0.5 | 10 | 10 | 0 | 10 | 11 | 0.5 | 10 | 11 | 0.5 | 11 | 11 | 0 | 14 | 14 | 0 | 12 | 13 | 0.5 | 11 | 11 | 0 |
| D3 | 8 | 7 | 0.5 | 9 | 9 | 0 | 10 | 9 | 0.5 | 8 | 9 | 0.5 | 10 | 10 | 0 | 12 | 11 | 0.5 | 12 | 12 | 0 | 10 | 10 | 0 |

| sBP | VM5 | | | | | | | | | | | | PM7 | | | | | | | | | | | |
| --- | --- | --- | --- | --- | --- | --- | --- | --- | --- | --- | --- | --- | --- | --- | --- | --- | --- | --- | --- | --- | --- | --- | --- | --- |
|  | S1  -v | S1  -v | SD | S2  -v | S2  -v | SD | S3  -v | S3  -v | SD | S4  -v | S4  -v | SD | S1  -v | S1  -v | SD | S2  -v | S2  -v | SD | S3  -v | S3  -v | SD | S4  -v | S4  -v | SD |
| D1 | 1.4 | 1.3 | 0.05 | 2.2 | 2.0 | 0.1 | 3.6 | 3.8 | 0.1 | 3.2 | 3.5 | 0.15 | 1.6 | 1.6 | 0 | 2.2 | 2.1 | 0.05 | 2.1 | 2.0 | 0.05 | 1.9 | 1.8 | 0.05 |
| D2 | 1.3 | 1.2 | 0.05 | 1.3 | 1.2 | 0.05 | 3.3 | 3.7 | 0.2 | 2.8 | 2.8 | 0 | 1.2 | 1.1 | 0.05 | 2.2 | 2.3 | 0.05 | 2.1 | 1.8 | 0.15 | 1.5 | 1.7 | 0.1 |
| D3 | 1.1 | 0.9 | 0.1 | 1.3 | 1.2 | 0.05 | 2.2 | 2.2 | 0 | 1.6 | 1.8 | 0.1 | 1.4 | 1.4 | 0 | 1.3 | 1.3 | 0 | 1.5 | 1.0 | 0.25 | 1.3 | 1.6 | 0.15 |
| sBP | S1  +v | S1  +v | SD | S2  +v | S2  +v | SD | S3  +v | S3  +v | SD | S4  +v | S4  +v | SD | S1  +v | S1  +v | SD | S2  +v | S2  +v | SD | S3  +v | S3  +v | SD | S4  +v | S4  +v | SD |
| D1 | 1.0 | 1.1 | 0.05 | 1.6 | 1.7 | 0.05 | 2.8 | 2.9 | 0.05 | 3.1 | 3.2 | 0.05 | 2.3 | 2.4 | 0.05 | 2.8 | 2.7 | 0.05 | 1.8 | 1.9 | 0.05 | 2.1 | 1.9 | 0.1 |
| D2 | 0.7 | 0.7 | 0 | 0.6 | 0.6 | 0 | 1.1 | 1.3 | 0.1 | 1.2 | 1.3 | 0.05 | 1.3 | 1.3 | 0 | 1.9 | 1.9 | 0 | 1.4 | 1.6 | 0.1 | 1.5 | 1.4 | 0.05 |
| D3 | 1.3 | 1.2 | 0.05 | 1.4 | 1.5 | 0.05 | 2.5 | 2.4 | 0.05 | 2.2 | 2.3 | 0.05 | 2.4 | 2.5 | 0.05 | 2.5 | 2.3 | 0.1 | 2.0 | 2.1 | 0.05 | 1.6 | 1.5 | 0.05 |

| BGE | VM5 | | | | | | | | | | | | PM7 | | | | | | | | | | | |
| --- | --- | --- | --- | --- | --- | --- | --- | --- | --- | --- | --- | --- | --- | --- | --- | --- | --- | --- | --- | --- | --- | --- | --- | --- |
|  | S1  -v | S1  -v | SD | S2  -v | S2  -v | SD | S3  -v | S3  -v | SD | S4  -v | S4  -v | SD | S1  -v | S1  -v | SD | S2  -v | S2  -v | SD | S3  -v | S3  -v | SD | S4  -v | S4  -v | SD |
| D1 | 0.18 | 0.17 | 0.005 | 0.22 | 0.21 | 0.005 | 0.22 | 0.22 | 0 | 0.22 | 0.24 | 0.01 | 0.24 | 0.29 | 0.025 | 0.27 | 0.24 | 0.015 | 0.29 | 0.28 | 0.005 | 0.24 | 0.21 | 0.015 |
| D2 | 0.20 | 0.18 | 0.01 | 0.20 | 0.19 | 0.005 | 0.28 | 0.29 | 0.005 | 0.25 | 0.25 | 0 | 0.17 | 0.16 | 0.005 | 0.27 | 0.25 | 0.01 | 0.27 | 0.23 | 0.02 | 0.24 | 0.22 | 0.01 |
| D3 | 0.15 | 0.13 | 0.01 | 0.13 | 0.15 | 0.01 | 0.19 | 0.19 | 0 | 0.16 | 0.18 | 0.01 | 0.19 | 0.18 | 0.005 | 0.19 | 0.21 | 0.01 | 0.22 | 0.17 | 0.025 | 0.19 | 0.16 | 0.015 |
| BGE | S1  +v | S1  +v | SD | S2  +v | S2  +v | SD | S3  +v | S3  +v | SD | S4  +v | S4  +v | SD | S1  +v | S1  +v | SD | S2  +v | S2  +v | SD | S3  +v | S3  +v | SD | S4  +v | S4  +v | SD |
| D1 | 0.18 | 0.18 | 0 | 0.21 | 0.22 | 0.005 | 0.26 | 0.23 | 0.015 | 0.26 | 0.26 | 0 | 0.21 | 0.22 | 0.005 | 0.25 | 0.25 | 0 | 0.27 | 0.30 | 0.015 | 0.26 | 0.24 | 0.01 |
| D2 | 0.15 | 0.16 | 0.005 | 0.16 | 0.16 | 0 | 0.17 | 0.19 | 0.01 | 0.19 | 0.20 | 0.005 | 0.25 | 0.25 | 0 | 0.30 | 0.30 | 0 | 0.28 | 0.32 | 0.02 | 0.28 | 0.27 | 0.005 |
| D3 | 0.18 | 0.16 | 0.01 | 0.17 | 0.17 | 0 | 0.20 | 0.22 | 0.01 | 0.22 | 0.23 | 0.005 | 0.25 | 0.25 | 0 | 0.30 | 0.29 | 0.005 | 0.31 | 0.32 | 0.005 | 0.29 | 0.28 | 0.005 |

| IBA | VM5 | | | | | | | | | | | | PM7 | | | | | | | | | | | |
| --- | --- | --- | --- | --- | --- | --- | --- | --- | --- | --- | --- | --- | --- | --- | --- | --- | --- | --- | --- | --- | --- | --- | --- | --- |
|  | S1  -v | S1  -v | SD | S2  -v | S2  -v | SD | S3  -v | S3  -v | SD | S4  -v | S4  -v | SD | S1  -v | S1  -v | SD | S2  -v | S2  -v | SD | S3  -v | S3  -v | SD | S4  -v | S4  -v | SD |
| 3-days | 58.2 | 57.3 | 0.45 | 60.5 | 59.5 | 0.50 | 33.2 | 32.3 | 0.45 | 36.2 | 35.5 | 0.35 | 26.9 | 27.3 | 0.20 | 23.1 | 23.4 | 0.15 | 26.3 | 26.0 | 0.15 | 22.3 | 21.8 | 0.25 |
| IBA | S1  +v | S1  +v | SD | S2  +v | S2  +v | SD | S3  +v | S3  +v | SD | S4  +v | S4  +v | SD | S1  +v | S1  +v | SD | S2  +v | S2  +v | SD | S3  +v | S3  +v | SD | S4  +v | S4  +v | SD |
| 3-days | 31.2 | 30.5 | 0.35 | 29.3 | 29.4 | 0.05 | 19.1 | 18.7 | 0.20 | 18.7 | 18.3 | 0.20 | 17.4 | 17.1 | 0.15 | 16.3 | 16.7 | 0.20 | 20.9 | 20.3 | 0.30 | 18.4 | 18.8 | 0.20 |

| IBR | VM5 | | | | | | | | | | | | PM7 | | | | | | | | | | | |
| --- | --- | --- | --- | --- | --- | --- | --- | --- | --- | --- | --- | --- | --- | --- | --- | --- | --- | --- | --- | --- | --- | --- | --- | --- |
|  | S1  -v | S1  -v | SD | S2  -v | S2  -v | SD | S3  -v | S3  -v | SD | S4  -v | S4  -v | SD | S1  -v | S1  -v | SD | S2  -v | S2  -v | SD | S3  -v | S3  -v | SD | S4  -v | S4  -v | SD |
| 3-days | 320 | 316 | 2.0 | 376 | 369 | 3.5 | 315 | 316 | 0.5 | 313 | 308 | 2.5 | 137 | 132 | 2.5 | 121 | 133 | 6.0 | 132 | 129 | 1.5 | 118 | 120 | 1.0 |
| IBR | S1  +v | S1  +v | SD | S2  +v | S2  +v | SD | S3  +v | S3  +v | SD | S4  +v | S4  +v | SD | S1  +v | S1  +v | SD | S2  +v | S2  +v | SD | S3  +v | S3  +v | SD | S4  +v | S4  +v | SD |
| 3-days | 129 | 127 | 1.0 | 122 | 123 | 0.5 | 117 | 123 | 3.0 | 116 | 113 | 1.5 | 89 | 88 | 0.5 | 84 | 84 | 0 | 79 | 73 | 3.0 | 74 | 75 | 0.5 |

| IBP | VM5 | | | | | | | | | | | | PM7 | | | | | | | | | | | |
| --- | --- | --- | --- | --- | --- | --- | --- | --- | --- | --- | --- | --- | --- | --- | --- | --- | --- | --- | --- | --- | --- | --- | --- | --- |
|  | S1  -v | S1  -v | SD | S2  -v | S2  -v | SD | S3  -v | S3  -v | SD | S4  -v | S4  -v | SD | S1  -v | S1  -v | SD | S2  -v | S2  -v | SD | S3  -v | S3  -v | SD | S4  -v | S4  -v | SD |
| 3-days | 70 | 63 | 3.5 | 85 | 81 | 2.0 | 95 | 99 | 2.0 | 87 | 90 | 1.5 | 35 | 34 | 0.5 | 40 | 41 | 0.5 | 47 | 39 | 4.0 | 32 | 33 | 0.5 |
| IBP | S1  +v | S1  +v | SD | S2  +v | S2  +v | SD | S3  +v | S3  +v | SD | S4  +v | S4  +v | SD | S1  +v | S1  +v | SD | S2  +v | S2  +v | SD | S3  +v | S3  +v | SD | S4  +v | S4  +v | SD |
| 3-days | 26 | 26 | 0 | 28 | 28 | 0 | 32 | 34 | 1.0 | 35 | 35 | 0 | 27 | 27 | 0 | 32 | 32 | 0 | 31 | 33 | 1.0 | 28 | 26 | 1.0 |

| IBB | VM5 | | | | | | | | | | | | PM7 | | | | | | | | | | | |
| --- | --- | --- | --- | --- | --- | --- | --- | --- | --- | --- | --- | --- | --- | --- | --- | --- | --- | --- | --- | --- | --- | --- | --- | --- |
|  | S1  -v | S1  -v | SD | S2  -v | S2  -v | SD | S3  -v | S3  -v | SD | S4  -v | S4  -v | SD | S1  -v | S1  -v | SD | S2  -v | S2  -v | SD | S3  -v | S3  -v | SD | S4  -v | S4  -v | SD |
| 3-days | 1163 | 1146 | 8.5 | 1211 | 1189 | 11.0 | 664 | 647 | 8.5 | 724 | 710 | 7.0 | 537 | 547 | 5.0 | 461 | 469 | 4.0 | 526 | 519 | 3.5 | 446 | 436 | 5.0 |
| IBP | S1  +v | S1  +v | SD | S2  +v | S2  +v | SD | S3  +v | S3  +v | SD | S4  +v | S4  +v | SD | S1  +v | S1  +v | SD | S2  +v | S2  +v | SD | S3  +v | S3  +v | SD | S4  +v | S4  +v | SD |
| 3-days | 624 | 610 | 7.0 | 587 | 588 | 0.5 | 381 | 374 | 3.5 | 375 | 367 | 4.0 | 348 | 341 | 3.5 | 323 | 333 | 5.0 | 419 | 406 | 6.5 | 368 | 377 | 4.5 |
